# Update of the human and mouse Fanconi anemia genes

**DOI:** 10.1186/s40246-015-0054-y

**Published:** 2015-11-24

**Authors:** Hongbin Dong, Daniel W. Nebert, Elspeth A. Bruford, David C. Thompson, Hans Joenje, Vasilis Vasiliou

**Affiliations:** Department of Environmental Health Sciences, Yale School of Public Health, 60 College St, New Haven, CT 06250 USA; Department of Environmental Health and Center for Environmental Genetics, University Cincinnati Medical Center, Cincinnati, OH 45267-0056 USA; HUGO Gene Nomenclature Committee (HGNC), European Bioinformatics Institute (EMBL-EBI), European Molecular Biology Laboratory, Hinxton, CB10 1SD UK; Department of Clinical Practice, University of Colorado Denver, Aurora, CO 80045 USA; Department of Clinical Genetics and the Cancer Center Amsterdam/VUmc Institute for Cancer and Immunology, VU University Medical Center, NL-1081 BT Amsterdam, The Netherlands

**Keywords:** Fanconi anemia, Genes, Classification, Human, Mouse, Genome

## Abstract

Fanconi anemia (FA) is a recessively inherited disease manifesting developmental abnormalities, bone marrow failure, and increased risk of malignancies. Whereas FA has been studied for nearly 90 years, only in the last 20 years have increasing numbers of genes been implicated in the pathogenesis associated with this genetic disease. To date, 19 genes have been identified that encode Fanconi anemia complementation group proteins, all of which are named or aliased, using the root symbol “FANC.” Fanconi anemia subtype (FANC) proteins function in a common DNA repair pathway called “the FA pathway,” which is essential for maintaining genomic integrity. The various FANC mutant proteins contribute to distinct steps associated with FA pathogenesis. Herein, we provide a review update of the 19 human FANC and their mouse orthologs, an evolutionary perspective on the FANC genes, and the functional significance of the FA DNA repair pathway in association with clinical disorders. This is an example of a set of genes––known to exist in vertebrates, invertebrates, plants, and yeast––that are grouped together on the basis of shared biochemical and physiological functions, rather than evolutionary phylogeny, and have been named on this basis by the HUGO Gene Nomenclature Committee (HGNC).

## Introduction

Fanconi anemia (FA) is an autosomal recessive disorder having an incidence of ~1/200,000–400,000 in most populations; the incidence is higher in Ashkenazi Jews (~1/30,000) and Afrikaners (~1/22,000) [1–4]. Most FA patients exhibit developmental abnormalities, developing early bone marrow failure and acute myelogenous leukemia (AML). Later, these patients display a higher risk of developing carcinomas of the head, neck, and anogenital region [5].

In recent decades, 19 human genes have been implicated in the causation of FA. These genes code for a group of proteins, viz. the Fanconi anemia subtype (FANC) proteins, which function cooperatively in a DNA damage recognition - and - repair pathway [6]. The FA pathway plays a crucial role in maintaining hematological homeostasis––particularly blood cell development and differentiation. FA is a congenital disease resulting from a somatic mutation in both alleles of the specific FANC gene; a notable exception is *FANCB* in which the mutation occurs on the X chromosome [7]. The primary causal factor in FA is generally thought to involve chromosomal instability in hematopoietic stem cells, a result of defective DNA repair caused by mutant FANC proteins [8, 9]. However, precise molecular mechanisms underlying the roles of specific FANC proteins (as well as their interactions with non-FANC proteins) in the FA pathway or other DNA damage repair pathways remain to be defined.

## Genetics of FA: human FANC genes

FA was first reported in 1927 by Swiss pediatrician Guido Fanconi. After finding three siblings who suffered from complex physical defects relating to aplastic anemia, Dr. Fanconi described the intrinsic nature of the abnormalities as “panmyelopathy” and proposed that multiple genes were likely causing the underlying complexity of FA symptoms [10]. It was later recognized that a high degree of underlying genetic heterogeneity existed in this disease [11]. Sixty-five years after the initial FA case was reported, the first FANC gene (*FANCC*) was identified, following development of a complementation-cloning method [12]. This approach was subsequently used in identification of an additional four FANC genes [13–16]. Through the use of complementation cloning, positional cloning, protein association, and candidate-gene and whole-genome sequencing, 19 FANC genes have now been identified as being involved in the etiology of FA (Table 1). The FANC genes are phylogenetically unrelated and have been grouped together and named or aliased with the FANC root symbol, based on association of mutations in the encoded proteins having a FA-like disease phenotype in combination with cellular hypersensitivity to DNA cross-linking agents.

**Table 1:** Human FANC genes

Gene symbol (NCBI gene ID)	Gene name	FANC symbol	Gene symbol synonyms	Chromosomal location	Ref seq RNA	Ref seq protein	Protein size (aa)
*FANCA* (2175)	Fanconi anemia complementation group A	*FANCA*	*FA*, *FA*-*H*, *FA1*, *FAA*, *FACA*, *FAH*, *FANCH*	16q24.3	NM_000135.2	NP_000126.2	1,455
*FANCB* (2187)	Fanconi anemia complementation group B	*FANCB*	*FA2*, *FAAP90*, *FAAP95*, *FAB*, *FACB*	Xp22.31	NM_001018113.1	NP_001018123.1	859
*FANCC* (2176)	Fanconi anemia complementation group C	*FANCC*	*RP11-80I15.2*, *FA3*, *FAC*, *FACC*	9q22.3	NM_000136.2	NP_000127.2	558
*BRCA2* (675)	Breast cancer 2	*FANCD1*	*RP11-298P3.4*, *BRCC2*, *BROVCA2*, *FACD*, *FAD*, *FAD1*, *FANCD*, *GLM3*, *PNCA2*, *XRCC11*	13q12.13	NM_000059.3	NP_000050.2	3,418
*FANCD2* (2177)	Fanconi anemia complementation group D2	*FANCD2*	*FA-D2*, *FA4*, *FACD*, *FAD*, *FAD2*, *FANCD*	3p25.3	NM_001018115.1	NP_001018125.1	1,451
*FANCE* (2178)	Fanconi anemia complementation group E	*FANCE*	*FACE*, *FAE*	6p21.22	NM_021922.2	NP_068741.1	536
*FANCF* (2188)	Fanconi anemia complementation group F	*FANCF*	*FAF*	11p15	NM_022725.3	NP_073562.1	374
*FANCG* (2189)	Fanconi anemia complementation group G	*FANCG*	*FAG*, *XRCC9*	9p13	NM_004629.1	NP_004620.1	622
*FANCI* (55215)	Fanconi anemia complementation group I	*FANCI*	*KIAA1794*	15q26.1	NM_001113378.	NP_001106849.1	1,328
*BRIP1* (83990)	BRCA1 interacting protein C-terminal helicase 1	*FANCJ*	*BACH1*, *OF*	17q23.2	NM_032043.2	NP_114432.2	1,249
*FANCL* (55120)	Fanconi anemia complementation group L	*FANCL*	*FAAP43*, *PHF9*, *POG*	2p16.1	NM_001114636.1	NP_001108108.1	380
*FANCM* (57697)	Fanconi anemia complementation group M	*FANCM*	*FAAP250*, *KIAA1596*	14q21.3	NM_020937.2	NP_065988.1	2,048
*PALB2* (79728)	Partner and localizer of BRCA2	*FANCN*	*PNCA3*	16p12	NM_024675.3	NP_078951.2	1,186
*RAD51C* (5889)	RAD51 paralog C	*FANCO*	*ROVCA3*, *R51H3*, *RAD51L2*	17q23	NM_002876.3	NP_002867.1	376
*SLX4* (84464)	SLX4 structure-specific endonuclease subunit	*FANCP*	*BTBD12*, *MUS312*	16p13.3	NM_032444.2	NP_115820.2	1,834
*ERCC4* (2072)	Excision repair cross-complementation group 4	*FANCQ*	*RAD1*, *XPF*, *ERCC11*	16p13.12	NM_005236.2	NP_005227.1	916
*RAD51* (5888)	RAD51 recombinase	*FANCR*	*BRCC5*, *HRAD51*, *HsRad51*, *HsT16930*, *MRMV2A*, *RECA*	15q15.1	NM_001164269.1	NP_001157741.1	340
*BRCA1* (672)	Breast cancer 1	*FANCS*	*BRCAI*, *BRCC1*, *BROVCA1*, *IRIS*, *PNCA4*, *PPP1R53*, *PSCP*, *RNF53*	17q21.31	NM_007294.3	NP_009225.1	1863
*UBE2T* (29089)	Ubiquitin- conjugating enzyme E2T	*FANCT*	*HSPC150*	1q32.1	NM_014176.3	NP_054895.1	197

Information about most published FANC gene mutations is available on the public Fanconi Anemia Mutation Database (http://www.rockefeller.edu/fanconi/). Genetic studies have revealed that mutations in the *FANCA*, *FANCC*, and *FANCG* genes are most common and account for ~85 % of FA cases [5, 17]. The *FANCA* gene was the second FANC gene identified [13]. *FANCA* mutations account for nearly ~65 % of FA cases [18]. About 200 different mutated *FANCA* alleles, comprising almost all the known mutation types, have been reported to date. Large intragenic deletions appear to be the major form of mutations [18].

*FANCC* mutations account for ~14 % of FA cases [5]. Among all known mutants, 322delG and IVS4+4A>T occurring in exon 1 and intron 4, respectively, are the most commonly observed *FANCC* mutations [19, 20]. Deletion of a single G at base 322 (322delG) produces a truncated protein of 44 amino acids. The IVS4+4A>T mutation results in either deletion of the entire exon 4 or a 40-bp deletion leading to a frame-shift.

*FANCG* was found to be identical to the X-ray repair cross-complementing protein 9 (*XRCC9*) gene; the gene is named as *FANCG*, with XRCC9 as an alias for the DNA repair protein. *FANCG* mutations account for ~10 % of FA cases [5] and have been implicated as founder mutations in different populations. For instance, a deletion mutation at c.637_643delTACCGCC was found to be associated with 82 % of FA cases in black populations of Southern Africa [21]. Screening of 45 FA families in Japan showed that nine of the families carried a splice mutation of IVS3+1G>C, with three of these nine families also carrying a 1066C>T mutation. Haplotype analysis revealed IVS3+1G>C and 1066C>T to be associated with Japanese and Korean ethnicities [22], respectively.

Mutations in the *FANCB*, *FANCD1*, *FANCD2*, *FANCE*, and *FANCF* genes, combined, account for ~13 % of reported FA cases [5]. The *FANCB* gene is the only FANC gene not to be autosomal, but rather X-linked. Point mutations, small insertions, and large deletions in the *FANCB* gene have been reported. For instance, a frame-shift mutation in exon 8 and a 3314-bp deletion in exon 1 (that includes some of the promoter region of *FANCB*) were reported in cell lines derived from FA patients [7]. Most *FANCB* mutations result in truncation of the encoded protein [23].

*FANCD1* is identical to the breast cancer susceptibility gene *BRCA2*; because the latter gene symbol was so extremely well-established, this gene is officially named *BRCA2* by the HGNC but has the alias of *FANCD1*. Studies reveal that FA-D1 patients have biallelic mutations in the *BRCA2* gene and express a truncated protein [24]. Heterozygotes in FA families (e.g., parents of FA patients) display increased risk of early-onset breast and ovarian cancers [25]. These findings connect FA and breast cancer, which share common mechanisms of defective DNA repair.

*FANCD2* encodes a protein that plays a central role in the FA pathway of DNA repair. *FANCD2* mutations involving large deletions and/or single-base changes have been reported [26, 27]. In addition, specific *FANCD2* mutations (for example, c.458T>C and c.2715+1G>A) have been associated with T cell acute lymphoblastic leukemia (T-ALL) and testicular seminoma [26, 27].

Mutations in the *FANCE* and *FANCF* genes account for 5–8 % of reported FA cases [5, 17]. Multiple types of mutation have been reported for *FANCE*, including a 355C>T transition in exon 2 in the EUFA410 FA cell line and the IVS5-8G>A splice mutation in EUFA622 cells [16, 28]. Disease-associated mutations have been reported throughout the single coding exon of the *FANCF* gene. The most commonly seen *FANCF* mutations are short deletions, resulting in frame-shifts and premature termination of the protein [29].

Mutations in the remaining FA genes, *FANCI*, *BRIP1* (*FANCJ*), *FANCL*, *FANCM*, *PALB2* (*FANCN*), *RAD51C* (*FANCO*), *SLX4* (*FANCP*), *ERCC4* (*FANCQ*), *BRCA1* (*FANCS*), and the recently reported *RAD51*(*FANCR*) and *UBE2T* (*FANCT*), contribute to <5 % of FA cases, combined [5, 17]. Documented mutations in these eleven genes––in FA patients and/or FA subtype cell lines––include point mutations, nucleotide insertions, splice-site mutations, and mutations resulting in protein truncation. For example, biallelic mutations [30] and a 30-residue C-terminal protein truncation [31] were reported for *FANCI*.

*BRIP1* (BRCA1-interacting protein C-terminal helicase 1) is the official gene symbol for *FANCJ*. Amino-acid substitutions in BRIP1/FANCJ, such as R251C, Q255H, and A349P, were found to be disease-causing [32, 33].

A dinucleotide insertion (c.755-756insAT) in *FANCL* [34], c.5569G>A transition in *FANCM* [35], and 3549C>G transition and 3549C>A transversion mutations in *PALB2*/*FANCN* [36] were reported in FA families. *PALB2* (partner and localizer of BRCA2) is the official gene symbol for *FANCN*. However, it should be noted that Lim et al. found individuals in the Finnish population who were homozygous for *FANCM* loss-of-function mutations but appeared to have no reported FA phenotype [37], casting doubt over the pathogenicity of this gene in FA.

*RAD51C* (RAD51 paralog C) is the official gene symbol for the *FANCO* gene. Meindl et al. reported 14 *RAD51C*/*FANCO* mutations––which included single-base-pair insertions, splice-site mutations, and sequence alterations [38]. *SLX4* (SLX4 structure-specific endonuclease subunit) and *ERCC4* (excision-repair cross-complementation group 4) are the official gene symbols for *FANCP* and *FANCQ*, respectively. Biallelic mutations in the *SLX4*/*FANCP* and *ERCC4*/*FANCQ* genes have been documented to cause FA subtypes [39–41].

*BRCA1* (breast cancer 1, early onset) has recently been identified as the 17th FANC gene and assigned the *FANCS* synonym [42]. Despite knowing that the BRCA1 protein cooperates in the FA pathway through forming a complex with several FANC proteins at DNA repair loci [43], *BRCA1* was previously precluded from being assigned as a FA gene due to the lack of reported biallelic *BRCA1* mutations in patients. However, recently, two cases of individuals harboring biallelic deleterious *BRCA1* mutations were reported [42, 44]. Detailed phenotypic and cellular characterization of one patient provided lines of evidence supporting the hypothesis that biallelic *BRCA1* (*FANCS*) mutations cause a new Fanconi anemia subtype associated with increased breast and ovarian cancer susceptibility [42].

Most recently, researchers have identified two new genes, mutations of which cause FA-like symptoms. The RAD51 gene encodes a key recombinase essential for homologous recombination of DNA during double strand break repair [45]. RAD51 is a mammalian homologue of the bacterial DNA repair protein RecA. A novel heterozygous mutation (T131P) in RAD51 was identified in a FA-like patient [46]. Expression of this dominant-negative mutant RAD51 in the patient’s cell line disrupted interstrand cross-link (ICL) repair but spared homologous recombination [46]. The authors proposed that RAD51 plays an ICL-repair-specific function through protection of nascent DNA strands from excessive processing at the ICL sites; this function may be regulated by BRCA2/FANCD1 [46]. The *UBE2T* gene encodes an ubiquitin-conjugating enzyme E2T that was already known to act in the FA pathway [47, 48]. Two unrelated individuals were found with biallelic *UBE2T* missense mutations that rendered the UBE2T protein unable to interact with FANCL [48]. The *RAD51* and *UBE2T* genes have now been given the aliases *FANCR* and *FANCT*, respectively.

## Evolution of *FANC* genes

**Fig. 1:**
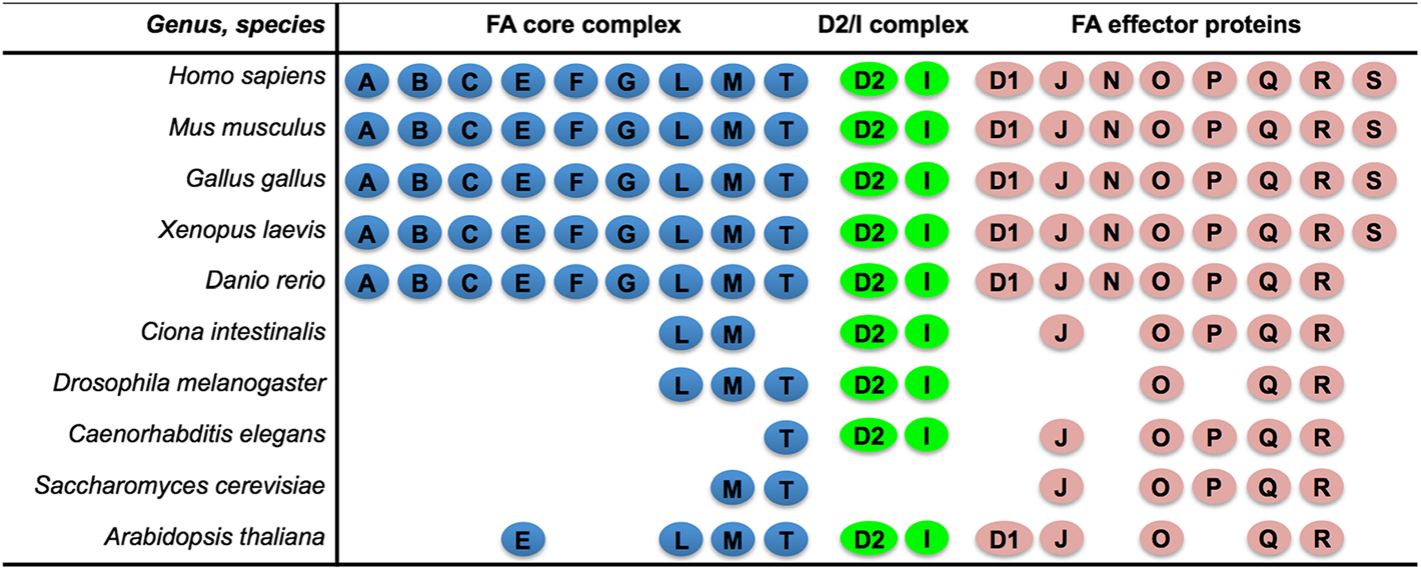
Overview of FA pathway genes identified in eukaryotic lineages. Representative species include mammals (*Homo sapiens*, *Mus musculus*, *and Gallus gallus*), amphibian (African clawed toad, *Xenopus laevis*), fish (zebrafish, *Danio rerio*), sea squirt (*Ciona intestinalis*), insect (*Drosophila melanogaster*), worm (*Caenorhabditis elegans*), yeast (*Saccharomyces cerevisiae*), and plant (*Arabidopsis thaliana*). *FANC* genes are grouped into three classes. Group I includes nine genes that encode proteins that form the FA core complex; group II encodes FANCD2 and FANCI that form the D2/I complex; group III comprises eight genes that encode FA effector proteins that function downstream of D2/I complex. Lower eukaryotes tend to be missing orthologues of the FA core complex genes. *A* = *FANCA*, *B* = *FANCB*, *C* = *FANCC*, *D2* = *FANCD2*, *E* = *FANCE*, *F* = *FANCF*, *G* = *FANCG*, *I* = *FANCI*, *L* = *FANCL*, *M* = *FANCM*, *D1* = *BRCA2*/*FANCD1*, *J* = *BRIP1*/*FANCJ*, *N* = *PALB2*/*FANCN*, *O* = *RAD51C*/*FANCO*, *P* = *SLX4*/*FANCP*, *Q* = *ERCC4*/FANCQ/*XPF*, *R* = *RAD51*/*FANCR*, *S* = *BRCA1*/*FANCS*, *T* = *UBE2T*/*FANCT*. If we extend this gene family update to include prokaryotes, it might be noted that, whereas no orthologs of any of the 19 eukaryotic *FANC* genes exist in prokaryote genomes, RAD51 (as a nineteenth FANC member in living organisms) qualifies as a homologue of bacterial RecA

It was originally thought that the FA DNA repair pathway was restricted to vertebrates. However, counterparts of several FA proteins have been discovered in lower eukaryotes, including sea squirt (*C. intestinalis*) [49], fly [50-52], worm [53-55], and yeast [56, 57], as well as in plants [58]. Nevertheless, it appears that the majority of lower eukaryotes possess a simplified FA pathway (Fig. 1) consisting of FANCL, FANCM, UBE2T, FANCD2, FANCI, BRCA2, BRIP1, RAD51C, SLX4, ERCC4, and RAD51. These FA proteins represent two components of the FA core complex, the D2/I complex, and most of the FA downstream effector proteins (*vide infra*).

What appear to have evolved later in the ancestral animal kingdom are most of the FA core complex proteins (Fig. 1). In addition to the 19 human FANC genes, two pseudogenes of the *FANCD2* gene have been annotated in the human genome, *FANCD2P1* (gene ID 100421239) and *FANCD2P2* (gene ID 101929530).

Comparative genomic analysis revealed genes of the FA pathway to be conserved among vertebrate genomes [49, 59]. A single ortholog of each of the 19 human FANC genes has been identified in other vertebrates––including rodents, chicken, *Xenopus*, and zebrafish (Fig. 1). However, the zebrafish genome has no *BRCA1* orthologue, suggesting that the entire vertebrate FA gene network had not been completely formed after the land-animal/sea-animal split.

In the mouse genome, 19 *Fanc* genes have been mapped to 13 chromosomes (Table 2). Mouse FANC proteins share 50–85 % sequence identity with their corresponding human orthologs. Transgenic knockout mouse lines have been generated for many *Fanc* genes [60-68]. In general, cells derived from *Fanc*(−/−) knockout mice recapitulate the phenotypes of human FA patient cells, whereas these mice only partially reproduce the clinical features of FA patients.

**Table 2:** Mouse Fanc genes

Gene symbol (NCBI gene ID)	Gene name	FANC symbol	Gene symbol synonyms	Chromosome location	Ref seq RNA	Ref seq protein	Protein size (aa)
*Fanca* (14087)	Fanconi anemia complementation group A	*Fanca*	*AW208693*, *Faca*	8	NM_016925.3	NP_058621.2	1,439
*Fancb* (237211)	Fanconi anemia complementation group B	*Fancb*	*RP23-32J17.2*, *BC022692*	X	NM_001146081.1	NP_001139553.1	853
*Fancc* (14088)	Fanconi anemia complementation group C	*Fancc*	*Facc*	13	NM_001042673.2	NP_001036138.1	558
*Brca2* (12190)	Breast cancer 2	*Fancd1*	*Rab163*	5	NM_001081001.2	NP_001074470.1	3,329
*Fancd2* (211651)	Fanconi anemia complementation group D2	*Fancd2*	*2410150007Rik*, *Au015151*, *Bb137857*, *Fad2*, *Fa4*, *Facd*, *Fad*, *Fancd*	6	NM_001033244.3	NP_001028416.2	3,329
*Fance* (72775)	Fanconi anemia complementation group E	*Fance*	*Rp24-68J3.2*, *2810451d06Rik*, *Ai415634*, *Aw209126*	17	NM_001163819.1	NP_001157291.1	1,437
*Fancf* (100040608)	Fanconi anemia complementation group F	*Fancf*	*A730016a17*	7	NM_001115087.1	NP_001108559.1	343
*Fancg* (60534)	Fanconi anemia complementation group G	*Fancg*	*Rp23*-*124l1.6*, *Au041407*, *Xrcc9*	4	NM_001163233.	NP_001156705.1	616
*Fanci* (208836)	Fanconi anemia complementation group I	*Fanci*	None	7	NM_145946.2	NP_666058.2	1,329
*Brip1* (237911)	BRCA1 interacting protein C-terminal helicase 1	*Fancj*	*Rp23-36p4.3*, *3110009N10Rik*, *8030460j03Rik*, *Bach1*, *Facj*, *Of*	11	NM_178309.2	NP_840094.1	1,174
*Fancl* (67030)	Fanconi anemia complementation group L	*Fancl*	*2010322C19Rik*, *AW554273*, *B230118H11Rik*, *gcd*, *Phf9*, *Pog*	11	NM_0253923.3	NP_080199.1	375
*Fancm* (104806)	Fanconi anemia complementation group M	*Fancm*	*Ai427100*, *C730036b14Rik*, *D12ertd364e*	12	XM_006515344.1	XP_006515407.1	2,021
*Palb2* (558923)	Partner and localizer of BRCA2	*Fancn*	*si:ch211-14k19.9*	7	XM_001919731.5	XP_001919766.3	1,104
*Rad51c* (114714)	RAD51 homologue C	*Fanco*	*RP23-209O5.3*, *R51H3*, *Rad51l2*	11	NM_001291440.1	NP_001278369.1	384
*Slx4* (850826)	SLX4 structure-specific endonuclease subunit homologue (S. cerevisiae)	*Fancp*	*Rai256635*, *Ai426760*, *Btbd12*, *D16bwg1016e*	16	NM_177472.5	NP_803423.2	1,565
*Ercc4* (50505)	Excision repair cross-complementing rodent repair deficiency, complementation group 4	*Fancq*	*Ai606920*, *Xpf*	16	NM_015769.2	NP_056584.2	917
*Rad51* (19361)	RAD51 recombinase	*Fancr*	*AV304093a*, *Reca*	2	NM_011234.4	NP_035364.1	339
*Brca1* (12189)	Breast cancer 1	*Fancs*	None	11	NM_009764.3	NP_033894.3	1812
*Ube2t* (67196)	Ubiquitin-conjugating enzyme E2T (putative)	*Fanct*	*2700084L22Rik*	1	NM_026024.3	NP_080300.1	204

## FANC complementation group proteins that cause the FA disorder

FA is characterized primarily by progressive bone marrow dysfunction and sensitivity to DNA cross-linking agents [5, 17]. This congenital disease results from loss-of-function of any of the 19 FANC genes, thereby revealing the essential roles of FANC proteins in maintaining chromosomal stability of hematopoietic stem cells. As noted above, more than 95 % of FA cases are attributed to mutations in known FANC genes [5]. In a few FA cases, genetic contributions remain unclassified. The unique clinical phenotype associated with *FANC* gene mutations implies that proteins encoded by these genes function in a common cellular pathway. This pathway, known as the FA/BRCA DNA repair pathway, functions pivotally in preserving genomic homeostasis in response to specific types of DNA damage [6].

**Fig. 2:**
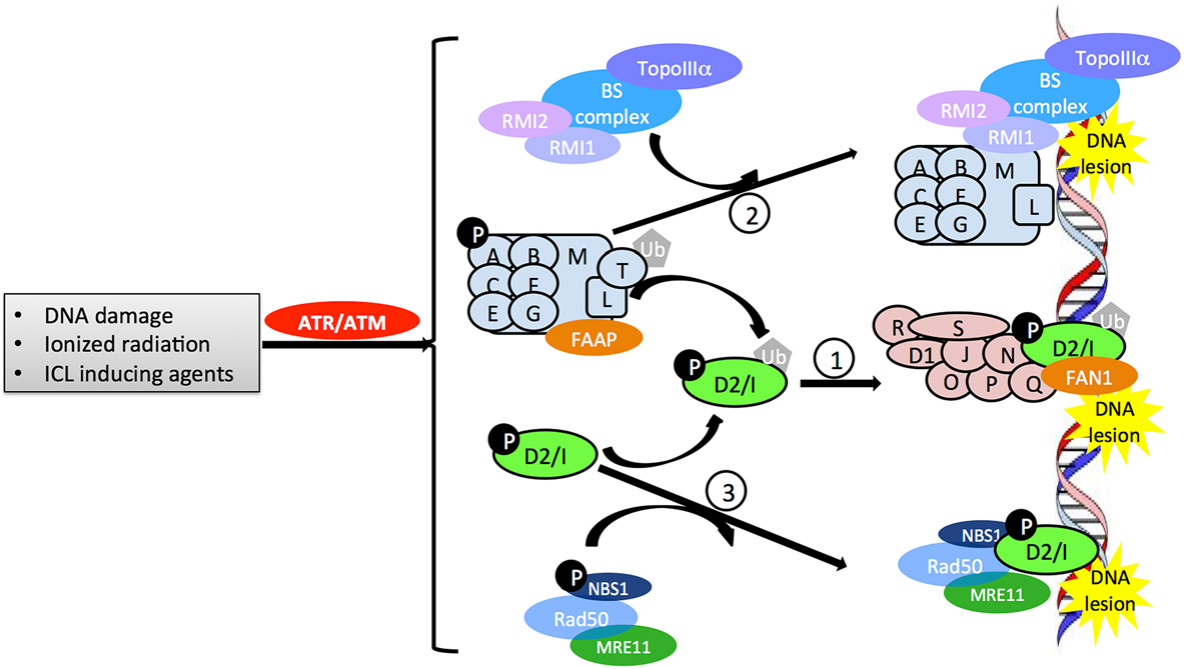
FA/BRCA pathway and crosstalk between FA and other DNA repair pathways. In response to upstream DNA damage signaling (such as phosphorylation by ATR/ATM), in the FA/BRCA pathway [1], the FA core complex forms––comprising FANCA (*A*), FANCB (*B*), FANCC (*C*), FANCE (*E*), FANCF (*F*), FANCG (*G*), FANCM (*M*), and FANCL (*L*) proteins, plus FAAP20, FAAP24, and FAAP100 (*FAAP*). This core complex binds UBE2T(T) via FANCL, which then activates FANCD2/I dimers through mono-ubiquitination. The activated FANCD2/I (D2/I) complex then translocates to DNA - damage sites and recruits downstream FA effector proteins––including BRCA1 (S), BRCA2 (D1), RAD51 (R), BRIP1 (J), PALB2 (N), RAD51C (O), SLX4 (P), and ERCC4 (Q), plus other DNA repair molecules (such as FAN1) to the lesion site to repair damage. In the FANCM/BS pathway [2], the FA core complex binds to the BS complex by way of interactions between FANCM-RMI1 and TopoIIIα of the BS complex and translocates to the lesion site. In the FANCD2/ATM pathway [3], in response to ionizing radiation or ICL-inducing agents, FANCD2 is phosphorylated by ATM and co-localizes with the NMR complex to repair DNA damage or cause S-phase arrest. *ICL* interstrand cross-links, *ATR* ataxia-telangiectasia-Ser/Thr-protein kinase, *ATM* ataxia telangiectasia mutated kinase, *FAAP* FA-associated proteins, *FAN1* FA-associated nuclease-1, *BS* Bloom’s syndrome protein, *RMI1/2* RecQ-mediated genomic instability protein 1/2, *TopoIIIα* topoisomerase IIIα, *NMR* the NBS1/MRE11/RAD50 complex

In a proposed model of the FA/BRCA DNA repair pathway (Fig. 2) [5, 17], eight FANC proteins––FANCA, FANCB, FANCC, FANCE, FANCF, FANCG, FANCL, and FANCM––plus three FA-associated proteins, FAAP20, FAAP24, and FAAP100, form the nuclear FA core complex. Fanconi anemia core complex associated proteins (FAAP) are functionally equivalent to FANC proteins, except that, thus far, no FA patients have been found whose disease phenotype could be ascribed to mutations in a FAAP-encoding gene. The FA core complex functions to receive upstream signals in response to DNA damage, through phosphorylation of multiple FA core units. The activated FA core complex binds the enzyme UBE2T (FANCT) via the putative plant homeodomain (PHD) zinc finger of the FANCL subunit, and then mono-ubiquitinates the FANCD2/I complex. This then translocates to DNA damage sites and recruits eight downstream FA effector proteins, i.e., BRCA1, BRCA2, BRIP1, PALB2, RAD51C, SLX4, ERCC4, RAD51, plus other DNA repair molecules, to DNA damage sites.

Distinct roles of FA proteins in the FA core complex and FANCD2/I complex have been suggested. FANCE contributes to core complex integrity––by promoting nuclear accumulation of FANCC protein and FANCA—FANCC complex formation and directly targeting FANCD2 for ubiquitination [69]. Phe-522, at the highly conserved carboxyl terminus of FANCE, was found to be a critical residue for mediating mono-ubiquitination of the FANCD2/I complex [70]. FANCL is an E3 ubiquitin ligase containing three WD40 repeats and a PHD zinc finger motif. These account for protein-protein interactions and recruitment of UBE2T (FANCT), respectively [71], both of which function importantly in FANCD2 mono-ubiquitination. FANCM in the FA core complex was reported to possess endonuclease and DNA helicase domains and is believed to function in translocation of the FA core complex along DNA [51]. FANCD2 mono-ubiquitination is essential for FA pathway-mediated DNA repair. However, the specific role of FANCD2 in the repair process remains unknown. A higher frequency of telomere dysfunction-induced foci (TIFs) and telomere sister-chromatid exchanges (T-SCE) was observed in primary cells derived from FA patients carrying a mutated *FANCD2* gene [72], suggesting its involvement in telomere regulation.

Of the 19 FANC proteins, some have been previously identified as part of different DNA damage repair pathways that may interact with the FA pathway [73, 74]. For example, FANCA has been reported to interact with DNA repair-associated proteins, such as the endonuclease ERCC4 and the ATP-dependent helicase SMARCA4 [75, 76]. In addition, FANCA mutant cells (derived from FA patients) exhibit defective mitochondrial respiration and impaired ATP production [77]. In human embryonic kidney (HEK 293T) cells, FANCA was found to influence centrosome integrity by way of its capacity to interact with NIMA-related kinase 2 (NEK2) [78].

### FA pathway combats genomic instability

One of the hallmarks of FA is hypersensitivity of cells from FA patients to the clastogenic and cytostatic effects of DNA cross-linking agents such as diepoxybutane (DEB) and mitomycin C (MMC) [5, 17]. In particular, DNA ICLs are highly cytotoxic and difficult to repair because these affect both strands of the DNA helix [79]. The FA/BRCA DNA damage-response pathway is a complex network that functions to remove ICLs through the coordinated actions of FANC proteins––plus other non-FANC proteins such as FAAPs and FANCD2/I-associated nuclease-1 (FAN1) [5, 17].

From an evolutionary point of view, the complete FA pathway is conserved in higher eukaryotes as an efficient pathway to manage ICLs [59]. In this model (Fig. 2), the FA core complex, comprising eight FANC proteins and three FAAPs, forms in response to DNA - damage signaling. The assembled FA core complex binds the ubiquitin-conjugating enzyme UBE2T via the FANCL subunit, which then activates the FANCD2/I complex via mono-ubiquitination at the K561 residue of FANCD2. The ubiquitinated FANCD2/I complex translocates to sites of DNA damage following binding with BRCA1/2 and RAD51, which ultimately triggers DNA repair.

Extensive crosstalk occurs between the FA/BRCA pathway and other DNA repair pathways. FANCD2-deficient cells are hypersensitive to ionizing radiation [80]. In these cells, the FANCD2 mutant cannot be efficiently phosphorylated by the Ser/Thr kinase ATM, resulting in a defect in the ionizing radiation-inducible S-phase checkpoint. FANCD2 was found to initiate S-phase arrest, in response to ionizing radiation, by interacting with ATM-phosphorylated nibrin of the MRE11–RAD50-NBN/NBS1 (MRN) complex [81, 82]. In response to ICL agents such as mitomycin C (MMC), phosphorylated FANCD2 co-localizes with the MRN complex at the DNA damage sites. This FANCD2/ATM pathway operates independently of the FANCD2/BRCA pathway because FANCD2 mono-ubiquitination-mediated formation of nuclear foci is not affected in ATM-deficient (*ATM(−/−)*) cells. In addition, a non-ubiquitinated mutant K561R of FANCD2 has no effect on FANCD2 phosphorylation, following ionizing radiation. FANCM was reported to interact with the Bloom syndrome (BS) complex using its highly conserved protein-protein interaction motifs, MM1 and MM2 [83]. The BS complex binds to the FA core complex by means of MM1-mediated FANCM-FANCF interaction and MM2-mediated interaction between FANCM, RecQ-mediated genomic instability 1 (RMI1), and topoisomerase IIIα (TOP3A) of the BS complex. This FANCM-mediated FA-BS crosstalk is required for MMC resistance of cells.

## FA and cancer

A high risk of carcinogenesis, particularly in hematopoietic and squamous cells, is another characteristic phenotype in FA patients, due to loss-of-function of FA proteins. FA-related malignancies vary between organs and cell types and in ages of onset and frequencies. Statistical analysis of 1300 identified FA cases revealed the highest frequency to be leukemia (9 %), followed by myelodysplastic syndrome (MDS) (7 %), solid tumors (5 %), and liver tumors (3 %) [84].

Using the International Fanconi Anemia Registry (IFAR) database to analyze 397 FA patients carrying *FANCC* mutations (specifically the IVS4 splice mutation in intron 4, 322delG or Q13X in exon 1 and R548X or L554P in exon 14) revealed that IVS4 and exon-14 mutations were highly correlated with severe congenital malformations and early onset of hematologic disorders at a median age of 2.7 and 2.1 years, respectively. Patients with exon-1 322delG or Q13X mutations exhibited mild congenital malformations and later onset of hematologic disease (median age 7.6 years) [85]. The IVS4 mutation caused distinct phenotypes in patients of Ashkenazi Jewish ancestry; interestingly, Japanese carriers display no significant clinical abnormality [86]. Patients carrying biallelic mutations in *FANCD1* demonstrate an early-onset leukemia (median age 2.2 years), compared with 13.4 years in other FA patients [87]. Increased risk of esophageal squamous cell carcinoma (ESCC) was reported to be associated with mutations in *FANCD2*, *FANCE*, *FANCL*, and *FANCA* in patients from an Iranian population [88]. Screening of germline DNA in 421 pancreatic cancer cases showed that, besides homozygote-associated cancer formation, heterozygous *FANCC* mutations were associated with increased susceptibility to pancreatic cancer [89]. A *BRIP1* mutation (c.2040_2041insTT), found at an allelic frequency of 0.41 % in an Icelandic cohort of 323,000 samples, was found to confer an elevated risk of ovarian cancer and is associated with an overall decrease of 3.6 years in lifespan––due to all cancers [90].

## Summary

Many of the currently identified 19 FA proteins have already been characterized with respect to their relative roles in FA etiology and FA-associated neoplasm. However, our understanding of the full range of molecular actions of these important and intriguing proteins remains to be clarified. For example, the specific role of individual FA protein members and their interaction with non-FA pathways in response to DNA damage are not fully understood. Reported cases of certain heterozygous *FANC* mutations, resulting in altered FA proteins, that predispose some individuals to cancer might involve non-FA mechanisms in specific organ or cell types. The observation that a specific *FANC* mutation can induce a different phenotype, depending on each patient’s specific genetic background, suggests that non-FA factor(s), or unidentified FA proteins, might also be participating in cancer risk. Further investigation of FA proteins should provide valuable insights into understanding molecular mechanisms involved in maintaining genomic integrity.

Finally, it should be mentioned that almost all previous Human Genomics “Nomenclature Update” reviews have emphasized gene nomenclature based on evolutionary divergence from an original ancestral gene. In the case of the *FANC* gene group––or “family”––we show here that the HGNC-approved classification of these 19 genes is based on a common phenotype resulting from a shared biochemical or physiological functions, rather than evolutionary divergence and, as such, this is not a classical “gene family.”
